# Sequence-Specific, RNA–Protein Interactions Overcome Electrostatic Barriers Preventing Assembly of Satellite Tobacco Necrosis Virus Coat Protein

**DOI:** 10.1016/j.jmb.2013.01.004

**Published:** 2013-03-25

**Authors:** Robert J. Ford, Amy M. Barker, Saskia E. Bakker, Robert H. Coutts, Neil A. Ranson, Simon E.V. Phillips, Arwen R. Pearson, Peter G. Stockley

**Affiliations:** 1 Astbury Centre for Structural Molecular Biology, University of Leeds, Leeds LS2 9JT, UK; 2 Division of Biology, Imperial College London, Sir Alexander Fleming Building, London SW7 2AZ, UK; 3 Research Complex at Harwell, Rutherford Appleton Laboratory, Harwell Oxford, Didcot, Oxon OX11 0FA, UK

**Keywords:** STNV, satellite tobacco necrosis virus, CP, coat protein, ssRNA, single-stranded RNA, TCV, turnip crinkle virus, VLP, virus-like particle, svAUC, sedimentation velocity analytical ultracentrifugation, TEM, transmission electron microscopy, PDB, Protein Data Bank, PS, packaging signal, smFCS, single-molecule fluorescence correlation spectroscopy, virus assembly, RNA–protein interactions, molecular mechanism

## Abstract

We have examined the roles of RNA–coat protein (CP) interactions in the assembly of satellite tobacco necrosis virus (STNV). The viral genomic RNA encodes only the CP, which comprises a β-barrel domain connected to a positively charged N-terminal extension. In the previous crystal structures of this system, the first 11 residues of the protein are disordered. Using variants of an RNA aptamer sequence isolated against the CP, B3, we have studied the sequence specificity of RNA-induced assembly. B3 consists of a stem–loop presenting the tetra-loop sequence ACAA. There is a clear preference for RNAs encompassing this loop sequence, as measured by the yield of *T* = 1 capsids, which is indifferent to sequences within the stem. The B3-containing virus-like particle has been crystallised and its structure was determined to 2.3 Å. A lower-resolution map encompassing density for the RNA has also been calculated. The presence of B3 results in increased ordering of the N-terminal helices located at the particle 3-fold axes, which extend by roughly one and a half turns to encompass residues 8–11, including R8 and K9. Under assembly conditions, STNV CP in the absence of RNA is monomeric and does not self-assemble. These facts suggest that a plausible model for assembly initiation is the specific RNA-induced stabilisation of a trimeric capsomere. The basic nature of the helical extension suggests that electrostatic repulsion between CPs prevents assembly in the absence of RNA and that this barrier is overcome by correct placement of appropriately orientated helical RNA stems. Such a mechanism would be consistent with the data shown here for assembly with longer RNA fragments, including an STNV genome. The results are discussed in light of a first stage of assembly involving compaction of the genomic RNA driven by multiple RNA packaging signal–CP interactions.

## Graphical Abstract

**Figure:**
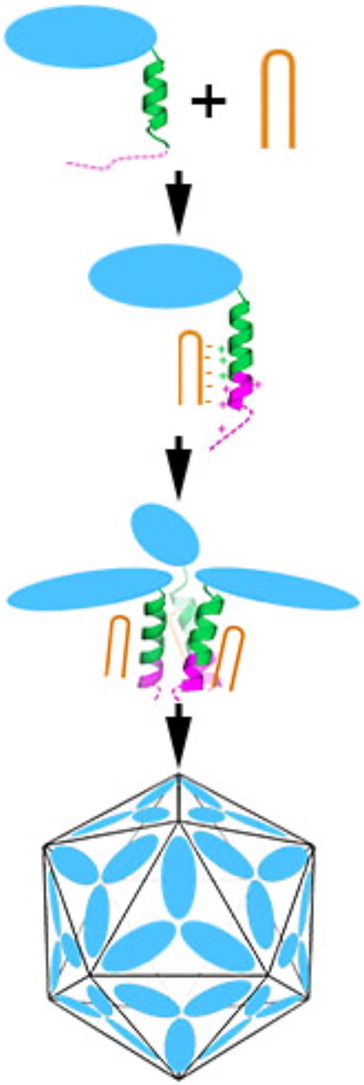

**Highlights:**

► Sequence specificity in satellite virus assembly. ► RNA-induced conformational change in a viral CP. ► RNA binding overcomes an electrostatic barrier to CP assembly. ► RNAs containing multiple preferred stem–loops assemble more efficiently. ► Evidence supports the existence of packaging signals in single-stranded RNA genomes.

## Introduction

Single-stranded, positive-sense RNA (+ ssRNA) viruses are major pathogens in every kingdom of life.^1^ The class includes infectious agents that are deadly to humans. Technical issues mean that it is highly unlikely that effective vaccines can be produced against the majority of them. This creates a need for an increased understanding of their fundamental structural biology in order to identify novel targets for antiviral therapy. Assembly of their capsids or nucleocapsids is an unexploited target.^2^ In these viruses, the protective protein shell often forms spontaneously in a co-assembly reaction with the viral RNA,^3^ creating two mechanistic problems.^4^ Firstly, how do they ensure selective packaging of their genomic RNAs in a milieu full of competing cellular RNAs? Secondly, for the majority of isometric capsids that display quasi-equivalence, how is the size and symmetry of the protective protein shell determined?^5^ These issues have been relatively neglected because in many examples, viral coat proteins (CPs) will self-assemble *in vitro* in the absence of RNA, or in the presence of non-cognate RNA or polyanions.^6,7^ The inference drawn until recently has been that assembly is dominated by the CPs and that the genomes are passive passengers in this process. Working with the model ssRNA virus, bacteriophage MS2, we have shown that this is far from the whole story.^8–12^ The MS2 genome has evolved to display multiple packaging signals (PSs) that help to switch the quasi-conformers of incoming subunits as assembly proceeds. At the same time, cognate CP binding promotes a collapse in the solution conformation of the genomic RNA. This reaction is dependent on multiple RNA–CP interactions that promote further protein–protein contacts to drive compaction. This two-stage mechanism ensures both specific encapsidation and faithful assembly of the capsid.^12^ These roles for the genomic RNA are consistent with the outcomes of *in vivo* infections, although they place additional evolutionary constraints, other than protein coding, on the genome sequence.

**Fig. 1:**
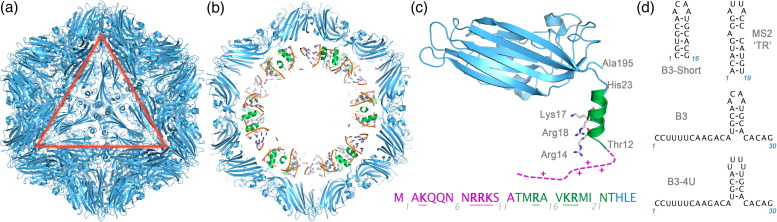
The components of the STNV system. (a) The STNV capsid. The X-ray structure of the recombinant STNV capsid shown as a blue cartoon (PDB entry 3S4G^14^). The view is along an icosahedral 3-fold axis, and the symmetry of the capsid is indicated by the red triangle, which corresponds to one face of the icosahedral particle. (b) A central (45 Å thick) slab through the STNV structure. The N-terminal helices are coloured green, and the structure of 7 nt per CP monomer of ordered RNA is also shown. (c) Close-up view of the STNV CP subunit. The subunit is a wedge-shaped jelly roll β-sandwich. The N-terminus is positively charged and visible in 3S4G up to the threonine at position 12. (Note that the amino acid numbering follows the PDB entries that begin with the genetically encoded N-terminal methionine as residue − 1). The unstructured portion (magenta) of the N-terminus contains a further four basic amino acid side chains as indicated in the sequence shown. (d) The sequences and predicted secondary structures of the oligoribonucleotides used in this study.

We have begun to study other examples of this class of virus to see which features of the assembly mechanism in the RNA phages are evolutionarily conserved and which are not.^13,14^ The plant satellite virus, satellite tobacco necrosis virus (STNV), is one such model.^15,16^ It is an example of a *T* = 1 capsid and is one of the smallest known viruses with a diameter of only 17 nm. A great deal is known about the virion structure (Fig. 1) and it was only the third virus to have its structure determined at atomic resolution.^17^ Its CP subunit has a Swiss-roll topology similar to that seen for the first two virus structures determined (tomato bushy stunt virus and southern bean mosaic virus)^4,18–20^ and now known to be present in the CPs of *Picornaviridae*. The N-terminal region of the protein is positively charged and forms an arm that is a common feature of many plant viruses, which is believed to interact non-sequence specifically with the viral RNA.^21^ The details of such interactions have not been seen in any previous virus crystal structures. In STNV, the density for the protein becomes disordered at the N-terminus beyond residue 12.^14,17,22^ In larger viruses requiring specification of quasi-conformers for their CP subunits, for example, the *T* = 3 tomato bushy stunt virus and turnip crinkle virus (TCV),^23^ a subset of the N-terminal arms form symmetry-related structural features that help to define the quasi-symmetry of the shell. Recent studies with TCV suggest that an additional role for the arms is proteolytic sensitivity in the expanded form, which is generated following cell entry.^24^ Such cleavages allow dissociation of a subset of CPs from a virus-like particle (VLP) structure that then extrudes its RNA from a unique site to interact with the host's translational machinery, thus avoiding silencing by the plant cell.^25^ Similar behaviour has been reported for the STNV virion,^26^ and we have shown that it also occurs with the mRNA VLP (unpublished results).

To investigate the roles of the N-terminal arms in assembly, and the molecular details of virion self-assembly, we used a recombinant version of the STNV-1 CP expressed from a synthetic gene in *Escherichia* *coli*. Expression results in assembly of *T* = 1 particles of the CP that are indistinguishable in the protein shell from the wild-type virion and that have been shown to encapsidate the mRNA.^13^ Unusually for any of the known satellite viruses,^15,27,28^ these particles are easily disassembled and reassembled *in vitro* by alterations in calcium ion concentration and pH. This *in vitro* reassembly protocol has allowed us to show that STNV CP collapses the conformation of its cognate genomic RNA, implying its assembly *in vivo* has similar features to the mechanism described above for MS2.^12^ Here, we use *in vitro* reassembly to probe the specificity of the CP interaction with genomic RNA and the roles of the CP arms in that process. We showed previously that CPs with N-terminal truncations of as few as 12 residues resulted in CPs that could not assemble.^13^ We were also able to use SELEX to isolate a preferred RNA binding motif for the CP. This is a short stem–loop structure with a loop sequence of AXXA, presented in 4-, 5-, or 6-base loops. Multiple copies (~ 30) of such degenerate sequence/structure motifs are present in the genomes of all three known STNV strains, consistent with a repeated RNA–CP interaction throughout the virion. This situation is analogous to satellite tobacco mosaic virus, where much of the genomic RNA appears to be packaged with pseudo-icosahedral symmetry and 30 segments of base-paired ssRNA are located along the particle 2-fold axes.^29–33^ Although we showed previously that isolated short oligonucleotides encompassing the aptamer B3 sequence trigger assembly *in vitro*, we did not establish whether this effect was sequence/structure specific, or whether the multiple copies of this motif within genomes are functionally significant.^13,14^ Here, we have explored the reassembly efficiency of STNV CP in the presence of different RNAs. These include B3 and sequence variants thereof, other short RNAs that present the AXXA motif on a different base-paired stem and longer RNAs including an STNV genome. The results show that there is sequence specificity for the AXXA motif, although it can be detected in a number of differing secondary structures. Other loop sequences still support assembly, albeit with markedly lower efficiency. Genomic RNA appears to be the most efficiently assembled longer RNA, implying that its multiple PSs are functional. The VLP reassembled in the presence of B3 crystallised, and its X-ray structure at 2.3 Å resolution was determined. Comparisons with the structures of the VLP containing a synthetic mRNA and the virion show that the preferred RNA PSs lead to increased ordering of the N-terminal arm, the α-helix extending by roughly one and a half turns. This conformational change includes residues 8–11, which are RKSA. Since the helices sit at 3-fold axes in the virion, this sequence-specific RNA–CP interaction has resulted in at least six positively charged residues coming into proximity. This may account for the inability of the CP to self-assemble in the absence of RNA and suggests that the capsomere may be a protein trimer. The positively charged N-terminus of many viral CPs is often assumed to be a nonspecific interaction domain that overcomes electrostatic repulsions within the RNA preventing encapsidation. For the STNV system, specific RNA interactions may actually overcome a similar barrier to assembly in the protein.

## Results and Discussion

### Sequence specificity of reassembly with short RNA stem–loops

To examine whether capsid assembly is sensitive to the preferred loop sequence motif derived by SELEX (AXXA), we prepared two 30-mer oligonucleotides encompassing variations on this motif (Fig. 1). These consisted of the B3 aptamer (B3) and an otherwise identical fragment (B3 4U) in which the 4-base loop sequence of B3 (ACAA) was replaced with UUUU. A truncated version of the B3 aptamer encompassing just the stem–loop region but stabilised by two additional base pairs (B3 short, 16 nt), as well as the known assembly initiation site for RNA bacteriophage MS2 (TR, 19 nt), which has a loop sequence of (AUUA),^34^ were also prepared. All oligonucleotides were synthesised, purified and characterised as described previously.^35^

**Fig. 2:**
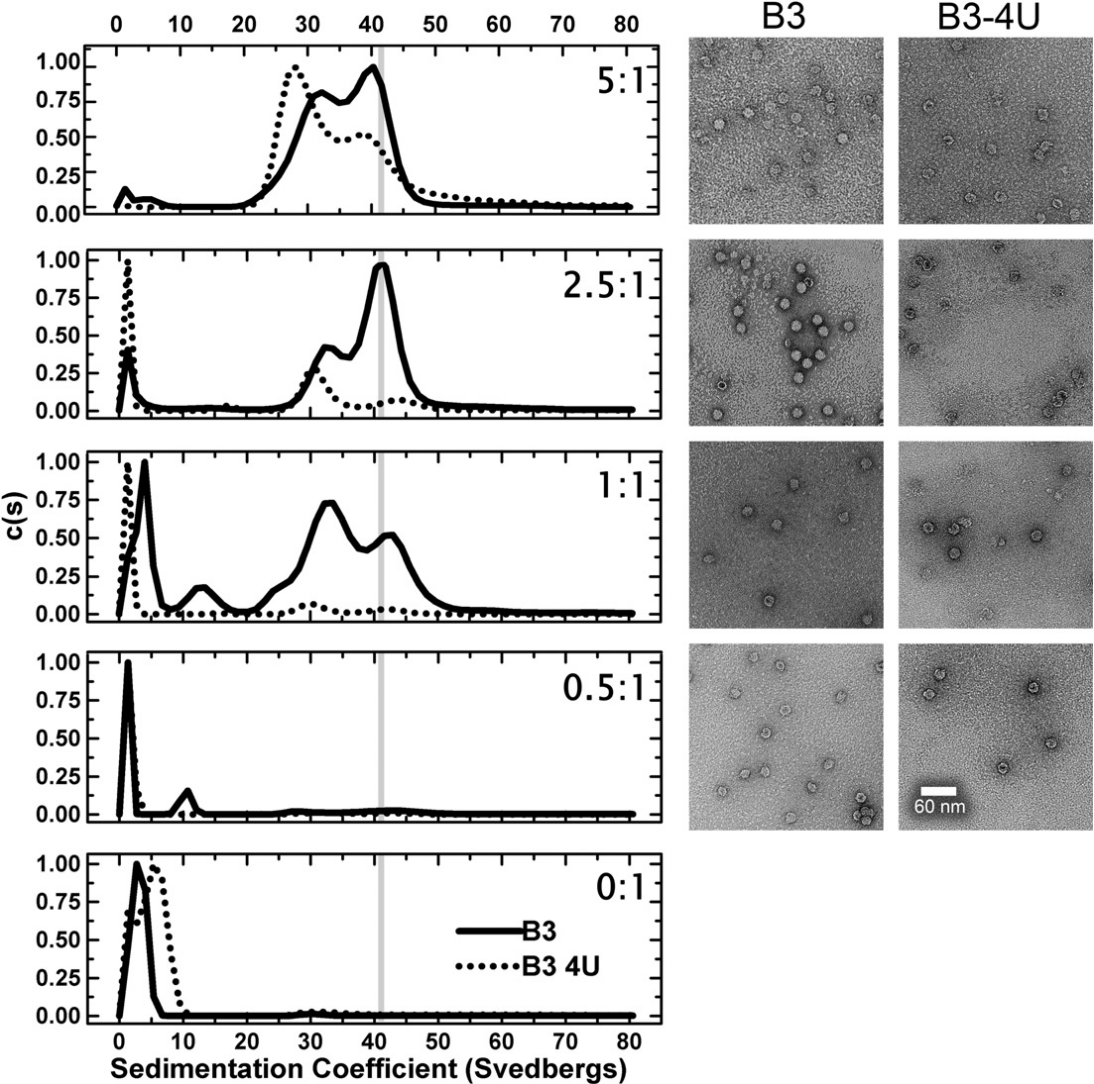
A comparison of STNV CP reassembly efficiency using B3 4U and B3 RNAs. Left panels show a titration of increasing molar ratios in reassembly reactions carried out as described in Materials and Methods analysed by svAUC. The corresponding images on the right are TEMs of the samples at the end of the reactions. The RNA concentrations were held constant at 2 μM and the molar ratios of CP:RNA are shown in each panel. The sedimentation coefficient of the recombinant VLP, here and throughout, is shown as vertical grey lines.

The efficiency with which each of these fragments promotes reassembly of the recombinant STNV-1 CP was then assessed using a standardised reassembly protocol.^13^ For RNAs ≤ 30 nt in length, CP was titrated into a fixed amount of RNA (2 μM) at a range of molar ratios 0.5:1 to 5:1, CP:RNA, spanning the range from sub-stoichiometric to excess protein with respect to capsid formation. Assembly of *T* = 1 VLPs occurred in this concentration range (Fig. 2). Higher ratios resulted in precipitation and/or aggregation and are not shown. The end products of assembly were analysed using sedimentation velocity analytical ultracentrifugation (svAUC) and negative-stain transmission electron microscopy (TEM). The percentage of assembled *T* = 1 capsid in each reaction was used as a measure of assembly efficiency. This was estimated from the area of the peaks in the sedimentation coefficient *c*(*S*) *versus* (*S*) plots (Table 1; see Materials and Methods). The distributions of material seen by svAUC reflect the particle content seen in the TEMs, suggesting that centrifugation did not disrupt any of the assembled complexes formed.

The first assay compared the B3 aptamer with its mutated version B3 4U (Fig. 2). The data are displayed as *c*(*S*) *versus* (*S*) plots. Under the same buffer conditions, the recombinant STNV VLP has a sedimentation coefficient of ~ 42 S. B3 is clearly more efficient at triggering capsid assembly than the oligonucleotide lacking the AXXA loop motif (Table 1; Fig. 2), promoting formation of VLPs even at the lowest molar ratio. At the 1:1 ratio, it is clear that assembly produces two distinct species, one sedimenting similarly to the *T* = 1 capsid and an intermediate sedimenting at ~ 28 S. The latter appears to be on the pathway to capsid assembly because at higher molar ratios, it decreases in amount as *T* = 1 capsid increases. TEM images show that these species correspond to intact *T* = 1 capsids (40 S) and incomplete shells with the dimensions compatible with a *T* = 1 architecture (28 S) (Fig. 2), respectively. In contrast, the sequence lacking the AXXA motif appears much less able to stimulate assembly at a 1:1 ratio and is significantly less efficient at a ratio of 2.5:1. The results confirm the dependence of assembly on RNA–protein interactions and are fully consistent with the aptamer sequence motif being a preferred RNA binding sequence for the STNV CP and thus better able to trigger assembly.

**Fig. 3:**
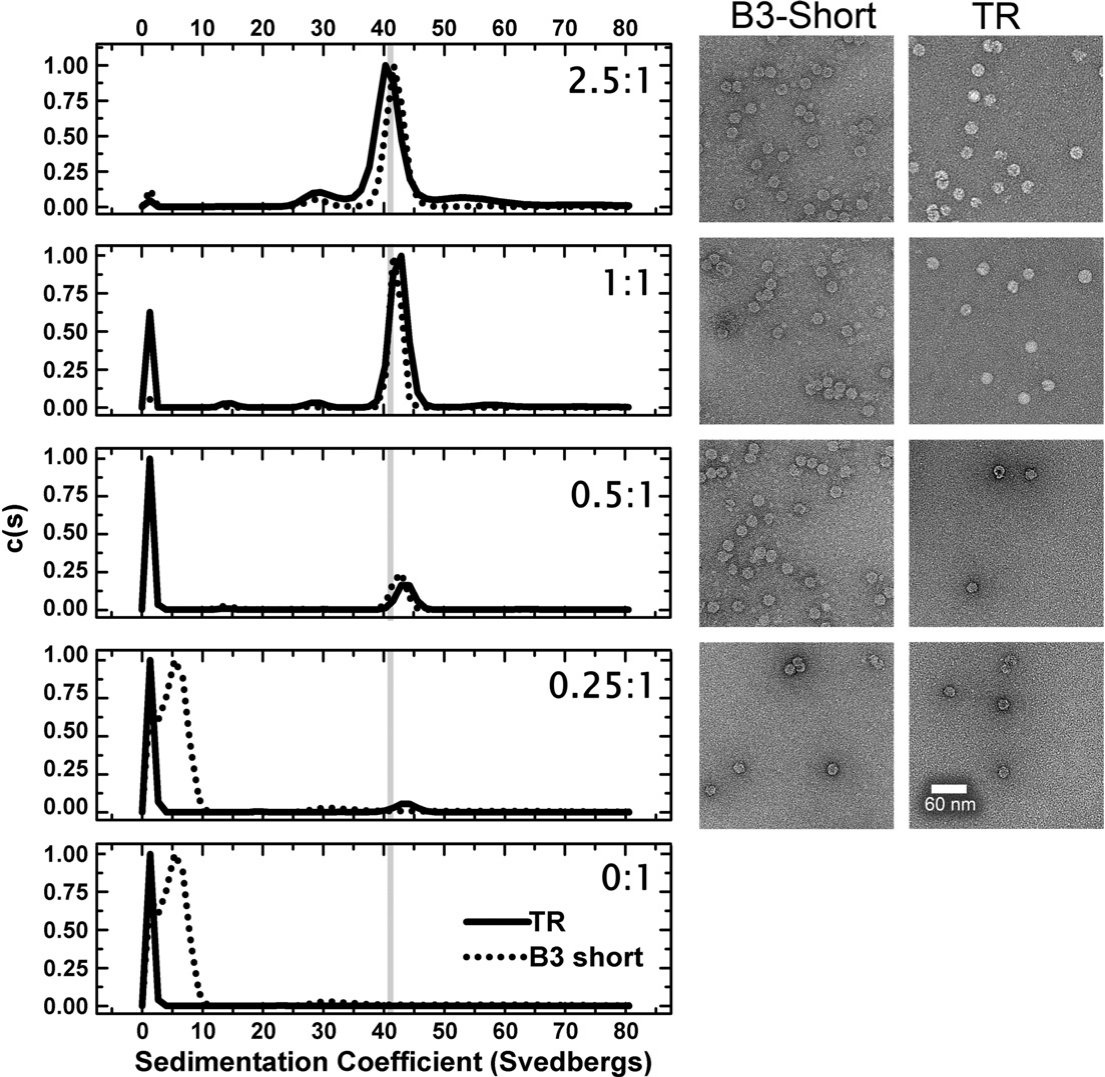
A comparison of STNV CP reassembly efficiency using B3 short and MS2 TR stem–loops. The RNA concentrations were held constant at 4 μM. All other details, here and in Fig. 5 and Supplementary Fig. 2, are as in the legend to Fig. 2.

B3 short and TR both have AXXA tetra-loop sequences. These are presented at the top of base-paired stems. The base-paired section of B3 has been extended by two G.C base pairs to increase its stability and the TR stem is interrupted by a single-stranded A on a fully base-paired stem (Fig. 1). In contrast to the loop sequence sensitivity seen for B3 and B3 4U, both the shorter stem–loops promote capsid formation with similar efficiencies at low CP:RNA ratios (Fig. 3; Table 1). Formation of intermediate species only seems to occur at the higher molar ratios. B3 short is significantly more efficient at promoting formation of capsid than B3 with roughly 90% of the material being *T* = 1 shells compared to only 50–60% for the larger RNA encompassing the same preferred binding motif. It is tempting to speculate that the flanking sequences in B3 are partially inhibitory of assembly.

### The N-terminal arm makes contact with the RNA

**Supplementary Fig. S1:**
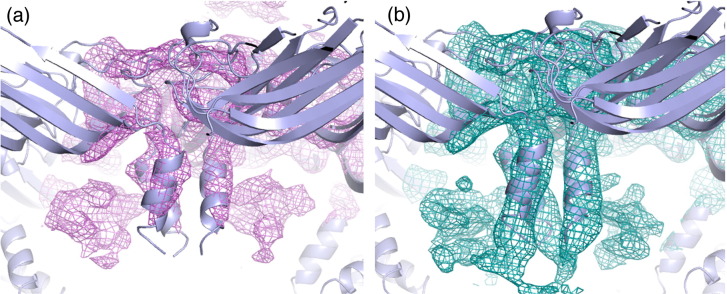
2*F*_o_ − *F*_c_ electron density for the STNV B3 complex showing the quality of the 60-fold averaged electron density, calculated using all data (217–2.3 Å) and the model fit. (a) is a 12-Å-thick slice through the capsid showing the clearly defined side chains as well as the presence of considerable non-protein internal electron density (electron density contoured at 0.038 e/Å^3^). (b) The calcium ion binding site at a capsid 5-fold axis and (c) at a capsid 3-fold axis. The electron densities in (b) and (c) are contoured at 5 σ.

**Fig. 4:**
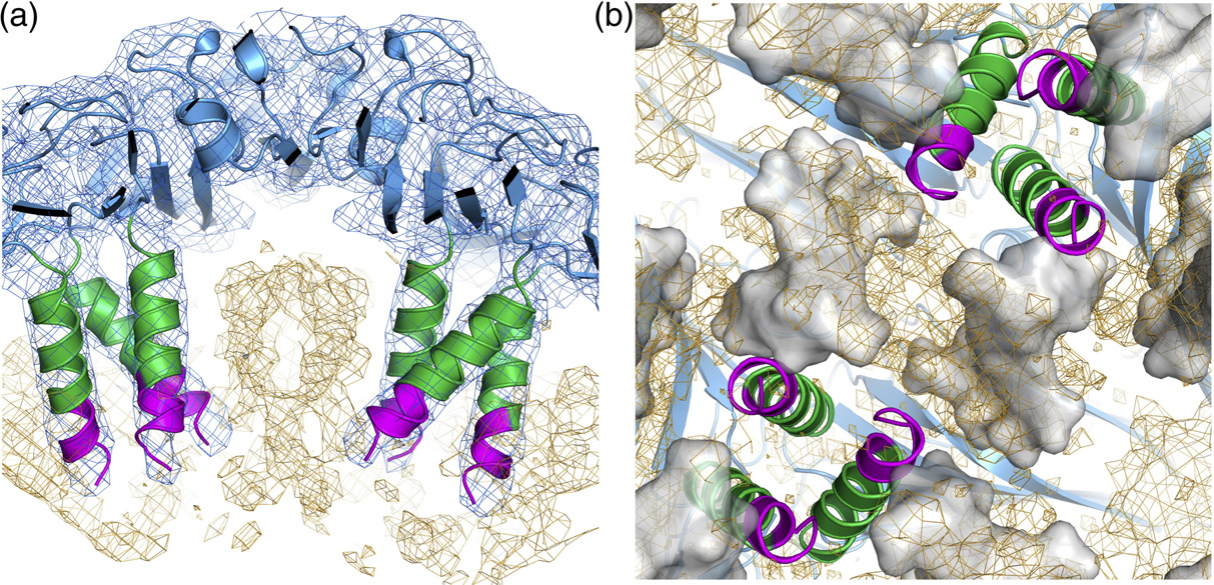
The X-ray structure of B3 VLP. (a) Ordered density in the N-terminal helices. The STNV CP is shown in cartoon representation and coloured as in Fig. 1. The orange mesh is the 60-fold averaged 2*F*_o_ − *F*_c_ electron density of the B3 VLP structure with the parts of the map corresponding to the CP shell masked away and contoured at 0.03 e/Å^3^. The blue mesh is the unmasked electron density contoured at ~ 0.07 e/Å^3^. The extra density at the N-terminal end of the helices is clearly visible, and residues 8–13 are shown in magenta. Residues 12 and 13 are present in other STNV structures but they adopt a different conformation in the mRNA containing VLP.^14^ (b) The B3 RNA density. The view is from the inside of the capsid, outward along an icosahedral 2-fold axis. The protein cartoon and orange mesh are as described in (a), while the grey surface is the solvent-accessible surface of the mRNA modelled in the study by Lane *et al.*^14^ (PDB entry 3S4G).

The reassembly reactions in the presence of B3, B3 short and TR were repeated on a larger scale, and the products were purified by size-exclusion chromatography. Each of the resulting VLPs had *A*_260/280_ values of ~ 1.7, similar to those of the recombinant VLP and the virion. These values confirm that the VLPs contain multiple copies of each RNA. For example, for B3, if each capsid on average contained ~ 30 RNAs each of 30 nt, then the RNA content would be ~ 75% of that of the virion (900/1239 nt) and 60 copies of the RNA would likely exceed the capacity of the particle. This might explain the observation that B3 is less efficient at promoting assembly than the short RNAs, perhaps because its flanking sequences occlude stem–loop binding sites. The B3 reassembled VLP (B3 VLP), but not the B3 short or TR equivalents, formed crystals that diffracted to 2.3 Å resolution (Table 2), allowing calculation of an electron density map (Fig. 4; Supplementary Fig. S1). The electron density map shown in Fig. 4 was calculated using only data between 217 and 6 Å in order to maximise the contribution of the low-resolution Fourier terms that contain information about the non-icosahedrally symmetric RNA. Supplementary Fig. S1 shows an overall view, as well as two closer views, of the quality of the map used for structure refinement (217–2.3 Å). The map contours are reported in e/Å^3^ as this is a more appropriate measure of the peak height than σ in this case, where maps are calculated with different regions masked out. As the RNA density in the B3 complex is not well defined, we have not attempted to model its interactions with the capsid. The specific interactions discussed below are derived from the mRNA VLP structure.^14^

**Supplementary Fig. S2:**
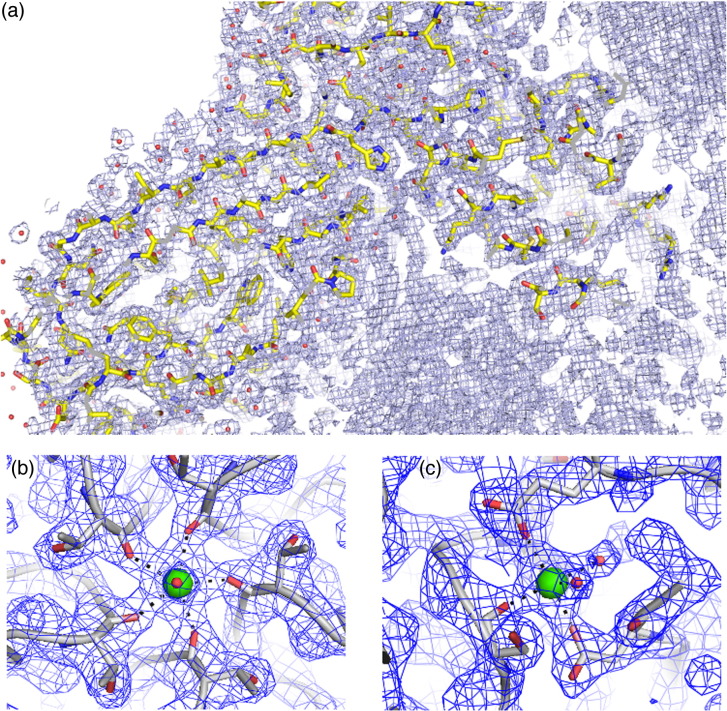
2*F*_o_ − *F*_c_ electron density for (a) the mRNA VLP^14^ and (b) the STNV B3 complex reported here. Both maps were calculated using data to a maximum resolution of 6 Å to enable clearer visualisation of electron density that does not follow the strict icosahedral symmetry of the capsid. Both maps are contoured at the same level. Additional electron density at the N-terminus, attributed to an extra turn of the helix, is clearly visible in (b).

The structures of the CP shell and subunits are very similar to those of previously published structures. The CP has a backbone r.m.s.d. of 0.138 Å compared to the virion [Protein Data Bank (PDB) entry 2BUK]^22^ and 0.271 Å compared to the VLP containing the mRNA (PDB entry 3S4G).^14^ The most variable region is the N-terminal helix of the CP, which has previously been described as the most flexible.^14^ The biggest difference between the B3 VLP structure and previously determined ones is that here the N-terminus is more ordered, permitting the formation of an additional turn at the N-terminus of the helix (residues 8–11 RKSA). This difference is clear when the B3 and mRNA VLP maps at the same resolution (217–6 Å) are compared (Supplementary Fig. S2). This conformational change suggests that sequence-specific binding of B3, which is most likely to occur with the globular body of the CP subunit, may orient the RNA stem such that the phosphodiester groups help to overcome electrostatic repulsions between CP N-termini. A similar mechanism is likely to occur with longer RNAs displaying multiple AXXA motifs. However, due to the intrinsic asymmetry of those RNA sequences, the conformations at the differing N-termini cannot follow strict icosahedral symmetry, accounting for their apparent disorder in the previous STNV structures. This may also be the origin of the reduced ordering of the B3 RNA with respect to the protein shell. The flanking sequences may result in reduced occupancy adjacent to the N-terminal helix, leading to conformational variability. It appears that specific RNA–protein contacts lead to alterations in STNV CP conformation. We previously established a similar effect of RNA binding to the capsomere of bacteriophage MS2,^8,9,11,34,36,37^ although in that case, the RNA-induced conformational change sets the quasi-conformer of the CP dimer. The B3 VLP structure suggests that a similar effect occurs even for proteins involved in forming a *T* = 1 capsid.

There is strong electron density for the B3 RNA that encompasses the smaller region of RNA density observed in the mRNA VLP. There is also significant additional density along the particle 2-fold axes adjacent to the tips of the N-terminal helices (Fig. 4b). This might correspond to the remaining residues of the CP N-terminus or additional RNA but its location is consistent with having the same RNA sequence at every site. The additional ordering of the helix emphasises the fact that individual RNA stem–loops bridge between protein 3-folds. The electron density map is icosahedrally averaged and suggests that there is RNA density associated with every CP subunit. This cannot, however, be an accurate representation of the VLP composition because that would imply that the particle contains 60 copies of B3, equivalent to 150% of the genomic content. Some of the RNA binding sites must therefore be unoccupied.

### The effects of multiple PSs in long RNAs

Although capsid assembly *in vitro* can be triggered by a short RNA stem–loop, *in vivo* the virus packages a 1239-nt genome. We showed previously that this sequence for STNV-1 potentially contains up to 30 copies of stem–loops having the preferred (AXXA) motif, and there are similar numbers of these motifs in the other known viral strains.^13^ The synthetic mRNA packaged in *E*. *coli* is only 590 nt long. It lacks eight copies of this preferred binding site with respect to the genome,^13^ both because it is shorter and because its sequence was mutated to ensure high-level bacterial expression. To examine the possible cooperative effects of RNAs containing multiple stem–loops, we compared reassembly using either the mRNA or an entire genome, in this case STNV-C (1221 nt). In order to make the comparison of assembly efficiency between the different long RNAs easier, we set up reactions in which the concentration of phosphodiester groups (= nts) was fixed (~ 60 μM) and titrated in increasing concentrations of the CP (1–10 μM). Although the CP:phosphodiester ratio increases during the titration, at each protein concentration, there are the same number of negatively charged groups on RNA to interact with. This is an important parameter because of the effects of charge neutralisation inferred from the B3 crystal structure (Fig. 4).

**Supplementary Fig. S3:**
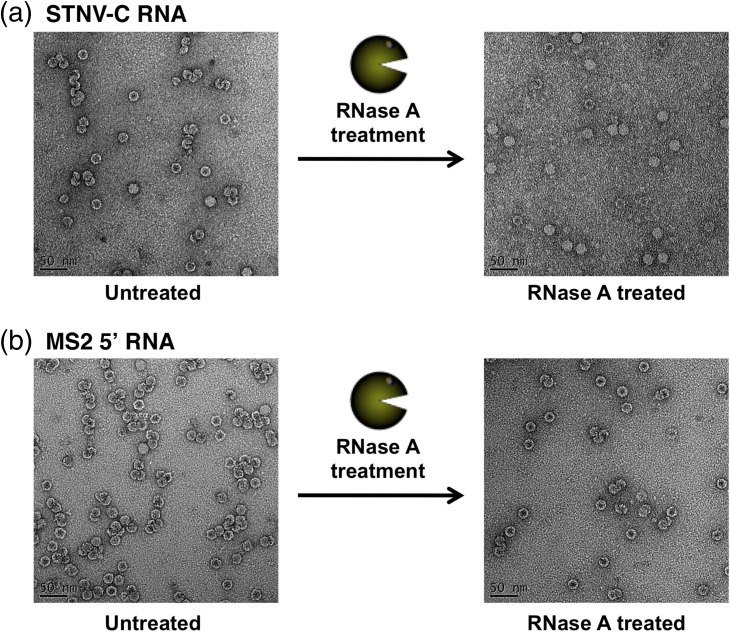
Reassembled STNV VLPs formed from STNV CP and either STNV-C RNA (Fig. 4) or the MS2 5′ RNA (Supplementary Fig. 4) at 5 μM [CP] appear aggregated (Fig. 4). Treatment of these particles with RNase A results in loss of the aggregates and the appearance of separated *T* = 1 particles. These results are consistent with the formation of fused capsids assembling on the same RNA.^7^

**Fig. 5:**
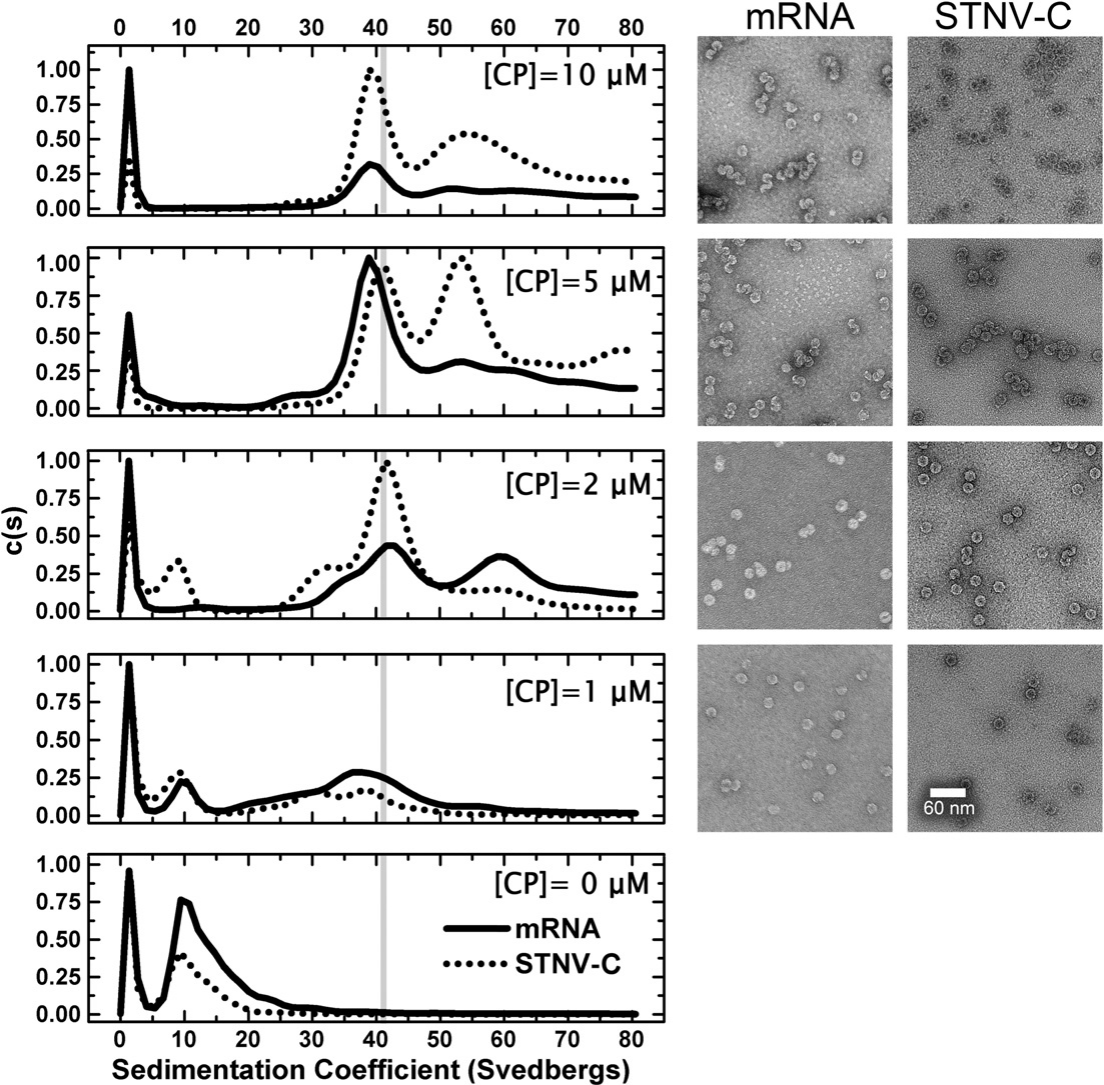
A comparison of STNV CP reassembly efficiency using STNV-C and STNV mRNAs. The RNA concentrations were as follows: mRNA, 100 nM and STNV-C, 50 nM, corresponding to ~ 60 μM phosphodiester concentration in each case. The CP concentrations are listed on the figure.

The efficiency and fidelity of *T* = 1 capsid formation are strikingly different for these two RNAs (Fig. 5). At the lowest CP concentration, both RNAs form species with a wide range of sedimentation coefficients. However, at 2 μM CP, the mRNA forms at least three species, one of which seems to correspond to the *T* = 1 capsid (42 S) with additional peaks at 60 S and 30 S. In contrast, under the same conditions, STNV-C RNA forms a majority of *T* = 1 capsids with a slower sedimenting shoulder, which may represent incomplete particles. Only a small fraction of input material sediments faster than 42 S. These results suggest that assembly is cooperative in the presence of longer RNAs, since the CP concentration is sub-stoichiometric with respect to formation of a capsid around every RNA. They also suggest that assembly with the mRNA transcript is less faithful than with the genome. At higher concentrations, the genome also shows evidence of a faster sedimenting peak, which becomes more dominant at the highest CP concentration. The TEMs associated with these reactions show evidence of closely associated particles, suggestive of the formation of fused double capsids. Subsequent treatment with RNase confirms this, the double shells falling apart post-treatment, suggesting that they are due to multiple capsid assembly initiation events on the same RNA (Supplementary Fig. 3).

It appears that a cognate genome is more faithful in *T* = 1 capsid assembly than a mutated shorter sub-fragment. This makes sense if the multiple preferred binding sites^12,13^ have sequences and physical locations within the RNA that facilitate the process. It is important to note that the CP subunits in these assembly reactions are from STNV-1 and so the genomic interaction with the STNV-C RNA is heterologous. However, both RNAs are capable of folding to present multiple copies of stem–loops displaying the preferred AXXA binding motifs in the loops.^12,13^

**Supplementary Fig. 4:**
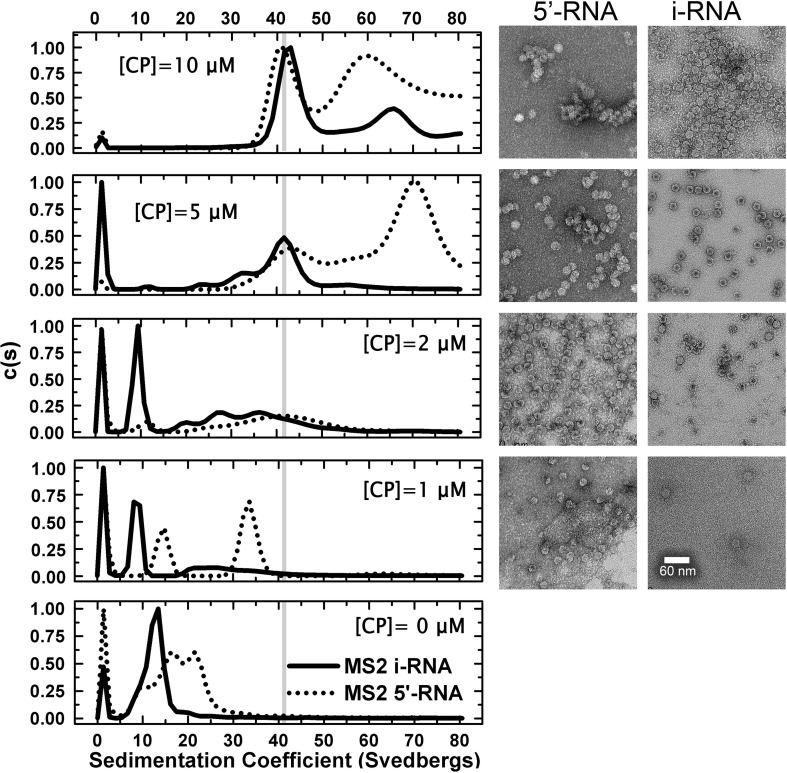
An overlay of STNV reassembly using MS2 iRNA and MS2 5′ RNA analysed by analytical ultracentrifugation (left) and the corresponding images from EM (right). The RNA concentrations were as follows: iRNA, 60 nM and 5′ RNA, 24 nM. The CP concentrations are listed on the figure.

Similar multiple putative PSs have recently been identified for MS2 CP in the MS2 genome using a combination of bioinformatics, structural and biophysical data (E. Dykeman, *et al.*, unpublished results). They are variations of the TR stem–loop oligonucleotides used here to trigger STNV assembly. The discrimination between genomes in assembly assays at single-molecule concentrations shown by their respective CPs re-emphasises the importance of both the existence of such sequences and their three-dimensional locations. This is especially true in light of the TR data described above. Many of the MS2 PSs contain a loop with an -AXXA- motif similar to TR, which triggers capsid assembly with STNV CP at the concentrations used here (Fig. 3). With this in mind, we examined *in vitro* packaging of MS2 genomic fragments by STNV CP (Supplementary Information, Supplementary Fig. 4). We compared assembly efficiency and fidelity with iRNA (927 nt), which is ~ 25% shorter than the cognate STNV genome, and 5′ RNA (2469 nt), which is roughly twice as large as the natural genome. Both these RNAs encompass the TR site. Remarkably, both RNAs support assembly of VLPs, some of which sediment at 42 S, consistent with the appearance of *T* = 1 particles by TEM, but only at CP concentrations several times higher than those that trigger assembly with the mRNA and STNV-C RNAs. For the shorter iRNA, the highest CP concentration yields mostly *T* = 1 capsids (Table 1), whereas the “oversized” 5′ RNA yields mostly faster sedimenting species. Brief treatment with ribonuclease released single particles from these large aggregates consistent with the view that they are the products of multiple capsid initiation events on a single RNA. Similar formation of multi-shelled structures when packaging “excess” RNA *in vitro* has been reported previously for cowpea chlorotic mottle virus.^7^ The results with the long RNAs are opposite to those seen with the short oligonucleotides in the sense that RNAs encompassing TR-like sites have different rank orders of triggering assembly, consistent with the view that there is a preference for ideally placed PSs within cognate genomes. This discrimination occurs over just a twofold protein concentration range under these conditions, emphasising the need to examine assembly at lower CP concentrations, where the reactions are not dominated by the high protein dependency.

### A molecular model of STNV assembly

**Fig. 6:**
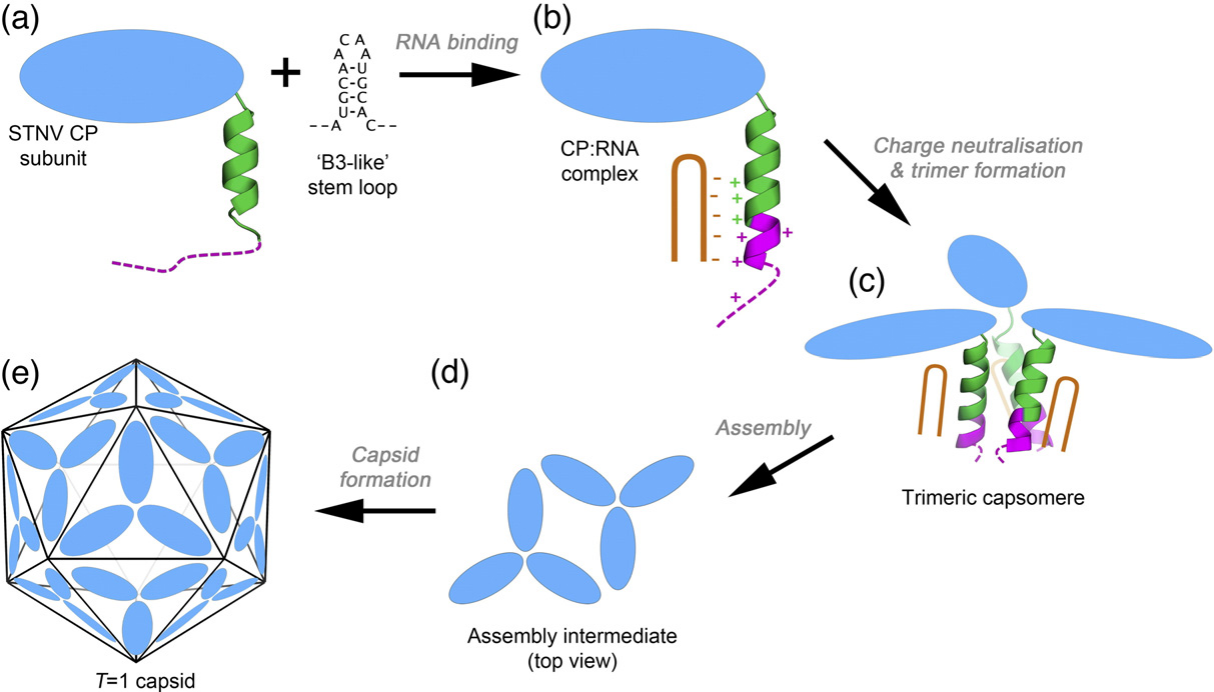
Schematic model of the STNV assembly process. (a) STNV CP subunits (blue oval) have an N-terminal extension that is partly helical (green) and partly unstructured (magenta). These extensions are highly positively charged with 7 Arg/Lys residues in the 24 residues at the N-terminus. (b) Binding of RNA stem–loops, exemplified by the B3 loop, at least partly neutralises the positive charge on the N-terminus, allowing the N-terminal helix to become longer and more ordered. (c) Once the N-terminal region has had its positive charge neutralised by RNA binding, the CPs can trimerize, forming an assembly-competent trimeric capsomere. (d) These trimeric capsomeres can then assemble to form higher-order structures and ultimately the *T* = 1 capsid (e).

Like many viruses having CPs with extended N-terminal arms, STNV assembly is dependent on those arms and truncation of the first 12 amino acids produces proteins that do not assemble.^13^ Many viral coat arms are rich in basic residues and it has long been assumed that they interact nonspecifically with viral RNA. The structural details of such interactions are unknown in any system to date. The results with STNV described above extend our understanding of these systems significantly and permit us to propose an assembly mechanism for the virus (Fig. 6).

In the presence of an RNA encompassing the B3 sequence, STNV CP assembles faithfully into a *T* = 1 shell, although also producing a smaller incomplete capsid product. This RNA–CP-induced reaction is sensitive to the loop sequence, since assembly is much less efficient with a UUUU motif. Although the details of the loop interaction with the globular body of the viral CP are not visible in the B3 electron density map, this interaction must position the RNA so that its stem lies adjacent to the N-terminal protein helices. It is then tempting to speculate that the most favoured orientation allows an additional section of the N-terminus to become ordered by neutralising the positively charged side chains in this region. These favourable electrostatic contacts would add to the interactions with positively charged amino acids (R14, K17 and R18) already identified in the mRNA VLP structure.^12^ Similar groups of charged amino acids are present in the CP sequences of STNV-2 and STNV-C, although in slightly different locations, implying that a similar mechanism for ensuring that capsid assembly is dependent on RNA binding may occur. The fact that the B3 sequence is less efficient than B3 short shows that assembly is also sensitive to the presence of additional RNA nucleotides outside the preferred stem–loop region. This may be a consequence of the limited volume within satellite viral capsids.^33^ The data presented here are consistent with the idea that far from simply providing charge compensation and hence allowing non-sequence-specific RNA binding,^7^ the N-terminal region plays a defined role preventing assembly in the absence of specific RNA sequences. The RNA facilitates overcoming an electrostatic barrier that could prevent trimer formation.

Given the increased ordering of the protein N-termini clustered around 3-fold axes in the presence of preferred RNA fragment sequences, the simplest assumption is that the primary capsomere is a trimer. Previously, we analysed the state of aggregation of RNA-free STNV CP by svAUC. The CP is a monomer with only traces of two higher-order species that could be trimer or pentamer.^13^ If preferred RNA binding promotes formation of the trimer, what promotes assembly of the *T* = 1 shell? The answer may be provided by the structures of the B3 and mRNA VLPs. Positively charged side chains from one subunit (R66, R91 and K123) are positioned so that they contact the phosphodiester backbone of the base-paired section of the stem–loop around the 3-fold axis of the neighbouring trimer. Thus, the RNA stem–loops bridge between trimers, as Fig. 4a illustrates.

The dominant paradigm for the assembly of ssRNA viruses is based on the idea that charge neutralisation between the genome and positively charged domains or faces of the CP subunits drive assembly.^38^ Such models are consistent with the observation that many non-cognate RNAs or even polyanions can trigger assembly of viral CPs *in vitro*. The assembly model for STNV described above is simply a variation on that theme. However, like the vast majority of assembly assays *in vitro*, the experiments reported here were carried out at relatively high concentrations of CP. *In vivo*, the CP concentration would steadily increase from zero during expression of viral genes. Under those circumstances, the protein would either begin to package genomic RNA immediately or remain as an unbound pool potentially interacting with other cellular RNAs and leading to inappropriate encapsidation.^12^ This is a dramatically different situation than most *in vitro* assays. The outcome of *in vivo* assembly reactions is usually a high yield of virions with the correct and complete protein architecture in which the cognate genome has been enclosed. How is such fidelity and efficiency achieved at low protein concentrations and in the presence of potential competitor RNAs?

Recently, we introduced single-molecule fluorescence correlation spectroscopy (smFCS) to monitor the conformation of viral RNAs during *in vitro* assembly of both STNV and MS2 RNAs. At smFCS concentrations of CP (100 nM *versus* the 10 μM used here), altered behaviour is observed. Both STNV and MS2 genomic RNAs show evidence for conformational ensembles in equilibrium in the absence of RNA. Most of these are too large to fit into the confined space of the virion. CP binding brings about a rapid conformational collapse so that it is small enough to fit. This collapse is the result of multiple CP–RNA and CP–CP interactions. The complex post-collapse is a partially formed protein shell of the correct size and symmetry, which then recruits additional CPs to complete the capsid. Non-cognate and non-viral RNAs do not result in collapse and trigger assembly. However, in contrast to the cognate interactions, these are very inefficient and produce significant numbers of misassembled structures. These data are not easily reconciled with a purely electrostatic assembly mechanism driven by the CP. They are however consistent with the idea that each viral RNA contains dispersed PSs, B3-like or TR-like for STNV^13^ or MS2 (E. Dykeman, *et al.*, unpublished results). Modelling the effects of such signals on assembly efficiency suggests that they are a more successful route towards high yields (E. Dykeman, *et al.*, unpublished results). PS affinity has a large electrostatic component rationalising previous reports of the importance of charge neutralisation.^7,38,39^ Since STNV and MS2 CPs have dramatically different topologies, the similarity in behaviour in the smFCS assays implies that there are conserved features in their assembly.

The results here demonstrate, at higher CP concentrations, the effects of PS recognition and the cooperativity that result from their appropriate placement within a genomic RNA. The level of discrimination between cognate and non-cognate interaction is, however, reduced under these conditions, leading to inappropriate recognition of TR and TR-like sequences in the MS2 RNAs. At smFCS concentrations, the cognate discrimination is very clear. These results explain the heterologous encapsidation widely reported for *in vitro* reactions.^3,6,40,41^ We have argued that by compacting genomic RNAs with a subset of the CPs required to form the completed capsid, viruses pay an early entropic penalty for assembly that they recoup via the reduced barriers involved in assembly with a condensed genome.^12^ The CPs bound in the compacted structure are stabilised by protein–protein as well as protein–RNA contacts, allowing cognate assembly to occur at low protein concentrations. These stabilised assembly intermediates would then compete effectively for CPs transiently bound to PS-like sites on cellular RNAs. In this way, genomic RNA assists both faithful encapsidation and assembly. Viral RNAs have been suggested to be more branched than other classes of RNA to facilitate this type of packaging.^42^ Even for viruses that are thought to encapsidate only nascent RNA strands emerging from the RNA-dependent RNA polymerase,^1,43^ these ideas are still valuable, because the RNA compaction required to enclose the genome in the capsid would be driven by the repeated RNA–CP contacts. Assembly with oversized or non-ideal genomes appears to happen with poor control over initiation events, leading to fused capsid structures that are resolved following RNase treatment (Supplementary Fig. 3). Such multiple shell formation during *in vitro* assembly has been seen before in more complex plant viruses such as TCV and cowpea chlorotic mottle virus.^7,44^

It is interesting to speculate whether the N-terminal regions in other ssRNA virus CPs, which are involved in specifying the CP quasi-conformers as well as interacting with their genomes,^4^ undergo similar conformational changes in response to binding preferred RNA sequences. Given the conservative nature of evolution, this is plausible, given the conservation of features of the assembly of both bacterial and plant viruses.^12^ A minimal PS has been defined *in vivo* for TCV consistent with this view^45^ and we are carrying out a similar analysis in that system. The results described above for STNV are entirely consistent with the newly emerging paradigm for this class of viruses, namely, that the genomic RNA plays a previously unsuspected role in ensuring faithful and efficient assembly.^2^

## Materials and Methods

### STNV CP purification

Recombinant STNV VLPs were purified according to Lane *et al*.^14^ STNV CP was purified by disassembling STNV VLPs in 50 mM Hepes and 10 mM ethylenediaminetetraacetic acid, pH 8.5, in the presence of Pepstatin A and Complete Protease Inhibitor Cocktail (Roche). STNV CP was separated from the mRNA by sequential Q-Sepharose FF anion exchange and SP-cation FF exchange chromatography. STNV CP was eluted from the SP-cation exchange column separately by a NaCl gradient. STNV CP was analysed by SDS-PAGE and the protein concentrations and *A*_260:280_ ratios were determined by UV spectrophotometry.

### RNA synthesis

Aptamer B3 sequence (5′-CCU UUU CAA GAC AUG CAA CAA UGC ACA CAG-3′), Aptamer B3 4U sequence (5′-CCU UUU CAA GAC AUG CAU UUU UGC ACA CAG-3′), B3 short sequence (5′-CGU GCA ACA AUG CAC G-3′) and MS2 TR sequence (5′-ACA UGA GGA UUA CCC AUG U-3′) were synthesised using *N*-benzoyl-protected adenosine, *N*-dimethylformamidinyl-protected guanosine and *N*-acetyl-protected cytosine; no protective group is required for uracil. The 2′ hydroxyl group was protected by *t*-butyldimethylsilyl groups and the 3′ was protected by 2-cyanoethyl-(*N*,*N*′-diisopropyl)-phosphoramidite (Link Technologies). Ammonia-saturated methanol was used to remove protecting groups and to cleave RNA from CPG resin at room temperature for 24 h. Methanol was removed under vacuum and the pellet was resuspended in anhydrous dimethyl sulfoxide. One volume of TEA.3HF was added and incubated at room temperature to remove *t*-butyldimethylsilyl from the 2′ hydroxyl groups. Each RNA was butan-1-ol precipitated and resuspended in DEPC-treated water (Severn Biotech), purified by ion-exchange HPLC and analysed by negative-mode electrospray ionisation mass spectrometry.

### *In vitro* transcription

Plasmids pETT22b-STNV^14^ and pUBS-STNV-C^46^ encoding STNV CP mRNA (590 nts) and STNV-C genome (1221 nt) were linearised by restriction enzymes HindIII and XhoI, respectively. MS2 iRNA (927 nt) and 5′ RNA (2469 nts) were transcribed from PCR templates.^11^ *In vitro* transcription from each template was performed using a MEGAScript *in vitro* transcription kit (Ambion) using the manufacturer's guidelines. All RNAs were purified by phenol:chloroform extraction and isopropanol precipitation. RNAs were analysed by 1% (w/w) agarose formaldehyde denaturing gel electrophoresis.

### STNV reassembly reactions

All RNAs were prepared initially at a concentration of 20 μg/mL and STNV CP was either omitted or added at defined molar ratios for RNAs ≤ 30 nt long, or the RNA concentrations adjusted to allow comparison of assembly at constant phosphodiester concentration, all in 500 μL final volumes. Each reassembly reaction was extensively dialysed in reassembly buffer (50 mM Hepes and 3 mM CaCl_2_, pH 7.5) overnight at 4 °C using 0.1- to 0.5-mL Slide-A-Lyzers (3 kDa molecular mass cutoff, Pierce).

### Analytical ultracentrifugation

Each sample (0.32 mL) was placed in a 1.2-cm pathlength two-sector meniscus-matching epon centrepiece cell constructed with sapphire windows. These samples were centrifuged at 15,000 rpm in an An50-Ti rotor in an Optima XL-1 analytical ultracentrifuge at 20 °C. Changes in absorbance at 260 nm of the solute were detected by absorbance optics, with a total of 100 scans being taken over approximately 12 h. Buffer densities and viscosities were calculated by Sednterp version 1.09.^47^ Radial absorbance profiles were fitted using the program Sedfit version 12.1b using a continuous distribution *c*(*S*) Lamm equation model.^48^

### Determining assembly efficiency of STNV VLPs

svAUC is used as a technique to determine the assembly efficiency of STNV VLPs. Previous work by Unge *et al.*^26^ shows that STNV VLPs sediment with a value of 28–48 S, dependent on the solution pH (8–5, respectively). The reassembly solution was pH 7.5; therefore, it was assumed that *T* = 1 VLPs would sediment at approximately 40 S, depending also on the size of RNA to be packaged. The sedimentation coefficient of the *T* = 1 mRNA VLP under these conditions was ~ 42 S. After continuous *c*(*S*) distribution analysis, peaks were integrated using size distribution of macromolecules as described by Schuck.^48^ The amounts of each sedimenting species are given as a percentage of the total material seen by absorbance scans in the analytical ultracentrifuge; Table 1 describes percentage-free RNA and material sedimenting as a *T* = 1 VLP.

### Electron microscopy

Each sample was added to a UV-irradiated Carbon/Formvar 300-mesh grid (Agar Scientific) and stained using 2% (w/v) uranyl acetate. Each reassembly reaction was analysed with either a Phillips CM-10 or a FEI Spirit G2 microscope at either 52,000 × or 49,000 × magnification, respectively.

### Production and purification of VLPs for X-ray crystallography

Recombinant STNV VLPs (~ 12 mg) were disassembled and STNV CP was purified by ion-exchange chromatography using the methodology described above. The total amount of CP was determined by UV spectrophotometry and sample quality was analysed by SDS-PAGE. Each RNA aptamer (B3, B3 short and TR) was added at a ratio of 5:1 (w/w, CP:RNA) before being extensively dialysed in reassembly buffer. Reassembled VLPs were then concentrated to 500 μL by Amicon Ultra® spin concentrators (Millipore) and purified by size-exclusion chromatography.

### Crystallisation

The VLPs were crystallised using the conditions previously established for recombinant STNV.^14^ Reservoir solutions contained 50 mM phosphate at pH 6.2–6.8, 0.2–0.8% (w/v) polyethylene glycol 6000 and 0–1 mM MgCl_2_. Drops were set up using 3 μL of VLP (7 mg mL^−^ ^1^) and 3 μL of reservoir solution. Diamond-shaped crystals appeared after 4 weeks of incubation at 25 °C and measured about 0.1 μm in their longest dimension.

Crystals were prepared for data collection by cryoprotection with glycerol before flash cooling in liquid nitrogen. Addition of glycerol was carried out in a stepwise fashion as follows: soaking for 60 s in 20% (v/v) glycerol, 80% (v/v) reservoir solution followed by a 20-s soak in 25% (v/v) glycerol, 75% (v/v) reservoir solution and finally a 1-s soak in 30% (v/v) glycerol, 70% (v/v) reservoir solution.

### Data collection

Diffraction data were collected at Diamond Light Source on beamline I02 at a wavelength of 0.9795 Å to a maximum resolution of 2.29 Å. High-quality low-resolution data were required to enable examination of the less ordered internal structure of the VLP. Three sweeps of data were therefore recorded from the same region of the crystal with varied detector and beamstop positions with resolution ranges of 217–7 Å, 116–3.8 Å and 30–2.29 Å. One hundred eighty degrees of data were recorded for each sweep with an oscillation angle of 1° for the very low resolution data and 0.5° for the medium- and high-resolution sweeps. All three sweeps of data were processed together using the 3dii option of the xia2 expert data reduction system (Table 1).^49^

### Phasing and refinement

The data were isomorphous with recombinant STNV VLP^14^ (PDB entries 1VTZ and 3RQV), and this structure was used to provide initial phases for map calculation. Rigid-body refinement of the entire capsid was carried out using REFMAC5.^50^ Sixty-fold averaged maps were calculated using Coot^51^ after which the CP structure was rebuilt and then refined, using strict 60-fold NCS, with REFMAC5 (Table 2).

### Examination of the capsid internal structure

Sixty-fold σ_A_-weighted 2*m*∣*F*_o_∣ − *D*∣*F*_c_∣ and *m*∣*F*_o_∣ − *D*∣*F*_c_∣ averaged maps were calculated using the Uppsala Software Suite.^52^ A mask comprising a single CP and the corresponding internal segment of the VLP was created using MAMA. Sixty-fold averaging of the mask was carried out using MAVE.

### Accession numbers

The coordinates and structure factors for the protein capsid in the presence of the B3 aptamer RNA have been deposited with the PDB with accession number 4BCU.

The following are the supplementary materials related to this article.

## Figures and Tables

**Table 1:** Assembly efficiency of STNV *T* = 1 capsids with different RNAs

RNA	[CP] 1 μM		[CP] 2 μM		[CP] 5 μM		[CP] 10 μM	
	%RNA	%*T* = 1	%RNA	%*T* = 1	%RNA	%*T* = 1	%RNA	%*T* = 1
B3	53.7	13.6	19.9	26.9	7.2	61.9	3.0	52.6
B3 4U	87.7	4.2	66.4	11.6	35.3	15.4	0.1	40.6
B3 short	83.1	14.8	57.3	31.6	2.7	92.9	4.0	86.7
TR	80.1	15.1	64.7	29.2	15.9	69.9	0.9	73.4
mRNA	11.4	35.0	1.3	23.0	0.5	37.3	0.0	25.3
STNV-C	18.4	19.9	7.4	54.1	0.0	23.1	0.0	27.7
MS2 i	39.3	0.0	26.2	*	0.0	50.5	0.0	41.3
MS2 5′	*	0.0	6.5	*	0.0	14.9	0.0	17.0

The percentages of free RNA or material sedimenting as a *T* = 1 VLP were determined as described in Materials and Methods. For the long MS2 RNAs, * indicates where the peaks of the *c*(*S*) *versus* (*S*) plots overlap, making it impossible to distinguish these components from other reassembly intermediates.

**Table 2:** X-ray data collection, processing and refinement statistics (values for the outer shell are given in parentheses)

Diffraction source	Diamond Light Source, I02
Wavelength (Å)	0.9795
Temperature (K)	100
Space group	*C*2
*a*, *b*, *c* (Å)	315.0, 300.6, 183.5
α, β, γ (°)	90, 94.4, 90
Resolution range (Å)	217–2.29 (2.35–2.29)
No. of unique reflections	756,410 (55,678)
Completeness (%)	99.6 (99.2)
Redundancy	5.5 (3.8)
〈*I*/σ*I*〉	9.3 (3.3)
*R*_pim_^a^ (%)	7.5 (38.6)
Resolution range (Å)	217.15–2.29 (2.35–2.29)
No. of reflections, working set	718,474 (52,951)
No. of reflections, test set	37,936 (2722)
*R*_cryst_^b^ (%)	15.5 (20.9)
*R*_free_^b^ (%)	19.8 (26.7)
No. of non-hydrogen atoms	100,140
Protein	88,140
Ligands	120
Water	13,440
Average *B*-factors (Å^2^)	18.4
Protein	16.9
Ligands	12.6
Water	28.2
r.m.s.d. bonds (Å)	0.023
r.m.s.d. angles (°)	1.683
Ramachandran plot	
Favoured regions (%)	96.8
Additionally allowed (%)	3.2
Outliers (%)	0

a $R_{\mathrm{pim}}=\frac{\sum_{h}\left(\sqrt{\frac{1}{n_{h}-1}}\right)\sum_{l}\left|I_{hl}-\langle I_{h}\rangle \right|}{\sum_{h}\sum_{l}\langle I_{h}\rangle }$

b $R=\frac{\sum \left|F_{o}-F_{c}\right|}{\sum \left|F_{o}\right|}$; *R*_free_ was calculated in the same way, but for the free set of reflections.
